# Tissue damage-induced axon injury-associated responses in sensory neurons: requirements, prevention, and potential role in persistent post-surgical pain

**DOI:** 10.3389/fpain.2025.1573501

**Published:** 2025-11-14

**Authors:** Kristofer K. Rau, Benjamin J. Harrison, Gayathri Venkat, Renée R. Donahue, Sara E. Petruska, Caitlin E. Hill, Bradley K. Taylor, Jeffrey C. Petruska

**Affiliations:** 1 Department of Anesthesiology, University of Louisville, Louisville, KY, United States; 2 Department of Anatomical Sciences and Neurobiology, University of Louisville, Louisville, KY, United States; 3 KY Spinal Cord Injury Research Center, University of Louisville, Louisville, KY, United States; 4 Department of Basic Science Education, Virginia Tech Carilion School of Medicine, Roanoke, VA, United States; 5 Biomedical Research Infrastructure Network (KBRIN), University of Louisville, Louisville, KY, United States; 6 College of Osteopathic Medicine, Biomedical Sciences, University of New England, Biddeford, ME, United States; 7 Center for Excellence in Neurosciences, University of New England, Biddeford, ME, United States; 8 Department of Physiology, University of Kentucky, Lexington, KY, United States; 9 Department of Ob/Gyn and Women’s Health, University of Louisville, Louisville, KY, United States; 10 Neural Stem Cell Institute, Albany, NY, United States; 11 Pittsburgh Center for Pain Research, Pittsburgh, PA, United States; 12 Department of Anesthesiology and Perioperative Medicine, Pittsburgh Center for Pain Research, and Pittsburgh Project to End Opioid Misuse, University of Pittsburgh School of Medicine, Pittsburgh, PA, United States; 13 Department of Neurological Surgery, University of Louisville, Louisville, KY, United States

**Keywords:** pain, electrophysiology, tissue damage, therapy development, inflammation

## Abstract

Pain resulting from tissue damage, including surgical incision, is often only partially responsive to anti-inflammatory drugs, suggesting the contribution of a neuropathic mechanism. Tissue damage leads to expression in dorsal root ganglion (DRG) sensory neurons of activating transcription factor 3 (Atf3), a known injury-induced transcription factor. Atf3 expression is associated with sensitization of cellular physiology and enhanced amplitude/duration of a nociceptive reflex. It is unclear how tissue damage leads to these changes in the sensory neurons, but it could include direct damage to the tissue-innervating axons and inflammation-associated retrograde biochemical signalling. We examined the necessity and sufficiency of incision, inflammation, and axonal conduction for induction of Atf3 in response to skin incision in rat. Incision outside of a single dermatome, but close enough to induce inflammation inside the dermatome, was not sufficient to induce Atf3 expression in the corresponding DRG. Incision inside the dermatome led to strong expression of Atf3. An anti-inflammatory drug did not prevent this induction of Atf3. In a mouse model of repeated injury – a major etiological factor for chronic pain – a second plantar incision induced a significant extension in the duration of mechanical hypersensitivity as compared to a single plantar incision. This corresponded with a remarkable increase in Atf3 expression in a rat model of repeated incision. Together, these results suggest that damage to axons innervating the skin is both necessary and sufficient for induction of Atf3 expression in sensory neurons. This is dramatically increased by repeated injury. Further, pre-treatment of the nerves innervating the incised skin with bupivacaine, a local anesthetic commonly used to reduce surgical pain, did not prevent induction of Atf3, indicating that conduction of action potentials is not necessary for induction of Atf3. Closure of incision with surgical glue or treatment with polyethylene glycol, known to enhance membrane integrity after injury, reduced incision-associated regulation of Atf3, Growth-Associated Protein-43 (*Gap43*), and electrophysiological changes. We conclude that tissue damage-induced pain arises from a mix of Atf3-independent inflammation-related mechanisms and axonal damage-associated mechanisms and therefore requires a mix of approaches to prevent/treat persistent post-surgical pain.

## Introduction

Pain associated with tissue damage, including surgery, is generally treated with anti-inflammatory drugs [e.g., (1–3)]. There is certainly a significant component of the pain, especially in acute and sub-acute phases, that is due to inflammation-induced sensitization of sensory neurons. However, in many cases the pain is only partially controlled by anti-inflammatory drugs and/or outlasts healing of the wound and resolution of inflammation. Etiologically, the most documentable and measurable form of long-lasting tissue damage-related pain is persistent post-surgical pain (PPSP). PPSP does not occur in all patients, but one study identified that moderate-severe pain occurs in 15%–30% of patients more than 2 years after surgery (4), and a recent review suggests 5%–85% (5), with rates varying by surgery type. A stronger mechanistic understanding of PPSP is needed.

Mechanistically, it is recognized that persistent pain “…is neither the result of an inflammatory process alone nor only the result of isolated injury to nerves” (6). This suggests that there are additional non-inflammation-related mechanisms at work, potentially including neuropathic mechanisms. The field acknowledges a neuropathic component to PPSP, in particular caused by overt injury to nerves. We hypothesized that covert neural damage in the form of injury to tissue-resident axons is another neuropathic component contributing to PPSP. Despite recognition of a clear role of various forms of nerve injury in the etiology of persistent post-surgical pain, the field appears apprehensive to expand this factor to recognize *axonal injury* as a contributing factor, evidenced by its absence in otherwise comprehensive reviews [e.g., (5, 7)]. Revising the contributing factor to be axonal injury, as opposed to nerve injury, would recognize the role of injury to sensory (and possibly autonomic and motor) neurons yet still encompass both injury to nerve tissue and peripheral target tissue.

The identification of nerve injury-like molecular and functional responses associated with tissue damage can offer new insights into the underlying causes of clinical pain, but it also raises many questions regarding necessary and sufficient conditions, particularly regarding common clinical practice. Further, if these responses are part of the overall pain experience, can they be prevented, reversed, or treated similarly to many inflammatory mechanisms, or are different treatments/preventions necessary?

Tissue damage can induce long-lasting changes in gene expression and physiology in sensory neurons that appear very similar to those induced by nerve injury. This was reported in models of skin incision (8–11), joint degeneration (12–14), dry eye (15), and therapeutic radiation treatment (16). We therefore sought to examine what clinically-modifiable factors might influence the expression of these injury-like responses in sensory neurons. We examined the influence of distance of injury from innervation zone, local anesthetics, anti-inflammatories, and closure methods on the expression of Activating Transcription Factor 3 (*Atf3*). Because repeated injury is often considered a risk factor for developing persistent pain, we also examined the impact of repeated injury on nociceptive behaviors and expression of *Atf3* and other pain-related genes.

Working from the principle that tissue damage can induce axolemma disruption, we also examined the effects of the “fusogenic” agent polyethylene glycol (PEG). PEG undoubtedly has many effects, but it is clear that it can induce sealing of damaged membranes, even to the extent of – at least temporarily – re-annealing/fusing the cut ends of axons, restoring conduction after nerve transection [e.g., (17–21)] and enhancing functional outcomes after spinal cord injury [e.g., (22–26)].

## Methods

### Animals

#### Rat

Animal care and procedures were carried out at the University of Louisville and were in accord with approved IACUC protocols. Age-matched adult female Sprague-Dawley rats (180–200 g; Taconic, Indianapolis, Indiana) were used for these studies. Rats were anesthetized with ketamine/xylazine (i.p. injection; 80 mg/kg ketamine; 10 mg/kg xylazine) and body temperature was monitored and maintained at 36°C throughout the surgeries which lasted 20–30 min. Puralube ointment (Dechra) was used to protect the rat's eyes during surgery. Following surgery, rats were given lactated ringer's solution (5 ml, i.p.) to prevent dehydration, and gentamycin (Gentafuse; 0.1 ml, i.m., every other day for 7 days) to prevent infection. Rats aged 4–5 weeks at the beginning of experiments were housed individually throughout the experiment.

#### Mouse

Mouse care and procedures were carried out at the University of Kentucky in accordance with approved IACUC protocols. Age-matched male and female C57BL/6J mice aged–7–9 weeks at the beginning of experiments were housed 4–5 per cage. All animals were monitored daily, maintained on a 14/10 h light/dark cycle at 20°C–22°C and 45% ± 10% relative humidity, with food and water provided *ad libitum*.

### Surgical procedures

#### Rat

Incisions involved cutting the full thickness of both the hairy skin and the underlying attached cutaneous trunci muscle. Following incision, the skin was closed with Ethilon nylon suture (5-0, Ethicon) or surgical staples and coated with Bacitracin antibiotic ointment (Actavis) to prevent infection. The experimental incision used to examine the molecular changes in the DRG neurons was made on the left side of the rats. It was located 1 cm lateral to the vertebral column and extended parallel to the vertebral column for 3 cm (including approximately the T7–12 dermatomes). The location of the incision ensured that the dorsal cutaneous nerves were not damaged by the incision. In experiments utilizing polyethylene glycol (PEG; 30% w/w in Ringer's solution; Sigma), 1 cc of PEG was applied post-surgically to the surface of the wound site. An additional 1 cc of PEG was injected IP.

#### Mouse

A plantar incision model (PIM) was conducted as initially described (27, 28). Mice were housed in a temperature-controlled room on a 14/10 h light/dark cycle and were provided food and water *ad libitum*. Mice were acclimated to the colony housing room for at least 4 d and then acclimated to handling for 3 min per day on each of 4 d before the initiation of the experiments. Briefly, postoperative hyperalgesia was induced by longitudinal incision of the skin and underlying plantaris muscle. Anesthesia was by isoflurane (5% induction and 1.5%–2% maintenance via a nose cone) and antisepsis by Chlorascrub then alcohol to the left hindpaw. A no. 11 scalpel blade was used to make a 5 mm long incision through the skin and fascia, beginning 2 mm proximal from the end of the heel and extending distally toward the digits. The underlying muscle was raised with curved forceps and then incised longitudinally, leaving the terminal connective tissue intact. The overlying skin was closed with synthetic 5-0 sutures (PDS* II, Ethicon), followed by application of antibiotic ointment. Sutures were removed on postoperative day 10. Sham controls received anesthesia but no surgical incision. In some experiments, additional groups included a second incision, as close to the original incision as possible.

### Behavioral test of mechanical sensitivity

Mice were acclimated to a temperature- and light-controlled room within individual Plexiglas bottomless boxes placed on the top of a stainless-steel mesh platform for 30–60 min before behavioral testing. Mechanical thresholds were assessed using an incremental series of eight von Frey filaments (Stoelting) of logarithmic stiffness (0.008–6 g). The 50% withdrawal threshold was determined using an up-down method (29). Each filament was applied perpendicular to the surface of the skin just lateral to the incision site with enough force to cause a slight bending of the filament. A positive response was defined by a quick withdrawal of the paw within 5 s. Gram force was logarithmically converted to 50% mechanical threshold. Thresholds were measured from baseline through 21 days after the first plantar incision to confirm the development and resolution of mechanical allodynia and then again after the second incision for 20 days. Control groups had corresponding single incision or sham incision. These studies were conducted at the University of Kentucky.

### Tissue collection

The range of time points examined (4–28 days post-incision) encompasses both sub-acute time points in which inflammation-related mechanisms occur, and later time points when inflammation has typically resolved (≥10–14 days). This range also encompasses the majority of the wound healing process. The skin is closed (surface wound contraction) to the point where sutures and/or staples can be removed by 7–10 days. Rats received an experimental incision and tissue was collected 4, 7, 14 or 28 days post-incision (DPI), or 4 days after a second incision with the first incision 14d or 28d previously. A control group of rats received no experimental incision. For tissue collection, rats were euthanized with an overdose of pentobarbital and transcardially exsanguinated with heparinized phosphate buffered saline (PBS; pH 7.4). This was followed by 33% vol/vol RNAlater (Qiagen) in heparinized PBS to help preserve the RNA. Three adjacent DRGs with projections to the incision site (typically T10–T12; innervation of incision site confirmed by gross-anatomical dissection) were collected, pooled together, and placed in 100% RNAlater overnight at 4°C and then stored at −80°C until RNA isolation.

### RNA isolation

To isolate RNA from the DRGs, samples were placed on ice and 350 μl RLT lysis buffer (Qiagen) and 2-mercaptoethanol was added. Tissue was homogenized for 1 min using a motorized dual Teflon glass homogenizer (Kontes). RNA was extracted using the RNeasy plus micro kit (Qiagen) as per manufacturer's protocol. Genomic DNA was removed using the DNA eliminator affinity spin column and RNA was purified by affinity purification using RNA spin columns. Samples were eluted in 14 μl of nuclease free water. RNA integrity was assessed by UV spectrometry and the Bioanalyzer (Agilent Technologies). RNA samples with 260 nm/280 nm ratios above 1.9 and 260 nm/230 nm ratios and RNA integrity numbers above 1.8 were used for RT-qPCR.

### RT-qPCR

RT-qPCR was used to quantify the expression of genes within DRGs following skin incision (Table 1). cDNA was generated from the RNA samples using the Quantitect first strand synthesis kit (Qiagen) according to the manufacturer's protocol. For each PCR reaction, 5 ng of cDNA template was used. Samples were run in triplicate, and control reactions (without template) were included with every amplification run. Primers mapped to exon boundaries within the consensus transcript. SYBR green RT-qPCR was carried out using a Rotorgene real time PCR detection instrument (Corbett Research). Relative fold-changes of RNA were calculated by the ΔΔCT method using *Gapdh* as the stable internal reference gene. Small differences in RT-qPCR reaction efficiency between primer sets were accounted for using the standard curve quantification methods.

**Table 1:** Sequences and characteristics for qPCR primers.

Gene	Forward primer (5′–3′)	Tm (°C)	Reverse primer (5′–3′)	Tm (°C)
*Gapdh*	ATGGCCTTCCGTGTTCCTAC	65.0	AGACAACCTGGTCCTCCTCAGTG	61.9
*Atf3*	GAGATGTCAGTCACCAAGTC	60.1	TTCTTCAGCTCCTCGATCTG	61.4
*Gap43*	CTAAACAAGCCGATGTGCC	63.2	TTCTTTACCCTCATCCTGTCG	62.9
*Scn3b*	GATTGAAGTCGTTGTCCCTG	61.0	CCCAGTAGATGAGCACTAGAG	61.2
*Cacna1g*	GGTCAATACACTCAGCATGG	60.1	CCGTAGACAAGCAGTTTCAG	61.0

### Tracer incision

For rats in which DRGs were to be examined electrophysiologically, an additional skin incision was made, on the right side, seven days prior to the experimental skin incision to allow for tracer injection and transport. This skin incision to enable tracer injection was placed on the right side to prevent injury to the axons of interest on the left side. Following incision on the right side, the skin was reflected to expose the underside of the contralateral (left) skin. 0.5% DiI (1,1′-dilinoleyl-3,3,3′,3′-tetramethylindocarbocyanine perchlorate; 5 mg FastDiI dissolved in 1 ml methanol; Invitrogen) was injected into the subdermal layer of the skin using a Hamilton syringe. Ten injections of 1 μl each, were used to target the terminal field as described previously (10). The experimental incision was approximately 5 mm distal to, and extended at least two dermatomes rostral and caudal from the DiI injected region. Preliminary studies indicated that this method maximized the percentage of DiI-labeled cells that were injured by incision, as indicated by Atf3/DiI co-localization.

### DRG dissociations for electrophysiology

DRGs were isolated, dissociated, and plated following previously published methods (30, 31). Enzymatic digestion of the DRGs was performed using dispase (neutral protease, 5 mg/ml; Boehringer Mannheim) and collagenase (type 1, 2 mg/ml; Sigma) in Tyrode's solution for 90 min at 35°C. To facilitate dissociation, the DRGs were gently triturated every 30 min. After enzymatic digestion, the cells were centrifuged at 800 rpm for 3 min, resuspended in fresh Tyrode's solution, and then plated onto poly-L-lysine (Sigma)-coated dishes. The dishes were kept in an aerated holding bath for at least 2 h before recording. Recordings were conducted within 10 h of DRG retrieval from the animal, a time frame that precedes the translation of Atf3 (32). Therefore, the observed Atf3 expression in dissociated/recorded neurons was attributed to the skin incision rather than the dissociation process.

### Electrophysiological recording

Whole-cell patch recording was used to determine the electrophysiological properties of individually isolated DiI-labeled neurons, specifically those projecting to the site of the skin incision. The electrophysiology procedures followed previously detailed methods (30, 31) and were conducted using a Scientifica SliceScope Pro system. Electrodes (2–4 MΩ) were prepared from glass pipettes using a horizontal puller (Sutter model P1000).

The extracellular solution (Tyrode's) consisted of 140 mM NaCl, 4 mM KCl, 2 mM MgCl_2_, 2 mM CaCl_2_, 10 mM glucose, and 10 mM HEPES, with the pH adjusted to 7.4 using NaOH. The recording electrodes were filled with a solution of 120 mM KCl, 5 mM Na_2_-ATP, 0.4 mM Na_2_-GTP, 5 mM EGTA, 2.25 mM CaCl_2_, 5 mM MgCl_2_, and 20 mM HEPES, also adjusted to pH 7.4 with KOH and with an osmolarity of approximately 315–325 mOsm.

DiI-labeled neurons were identified using brief illumination with epifluorescence microscopy (total exposure of field <1 min). Once identified, whole-cell recordings were conducted using an Axoclamp 2B (Molecular Devices). Stimuli were controlled, and digital records were captured using pClamp10 software and a Digidata 1440 (Molecular Devices). Series resistance (RS) was compensated by 50%–70%. Whole-cell resistance (RM) and capacitance were assessed using voltage transients associated with small step commands (10 mV) via pClamp software. All experiments were carried out at room temperature, and only cells with a resting membrane potential (RMP) of −40 to −70 mV were included.

After obtaining and stabilizing individual DRG neurons in whole-cell patch-clamp voltage-clamp mode, the cells were switched to current-clamp mode to assess changes in membrane potential. To evaluate cellular excitability, rheobase and action potential frequency in response to standardized depolarizing current steps were acquired. Action potentials were evoked with a 1 ms, 2 nA current step, and the average of ten action potentials was used to determine the after-hyperpolarization duration (80% recovery to baseline) and amplitude (voltage decrease from RMP to the lowest point of the after-hyperpolarization) as previously published (33). Action potential threshold and duration at threshold (APDt) were measured at the rheobase using 500-ms square pulses, increased in 50-pA increments every 2 s and refined further with 5-pA increments. APDt was measured from the first upward deflection of the action potential waveform to its return to the threshold membrane potential. The number of evoked action potentials and peak instantaneous frequency were determined using increasing voltage steps (1-second stimulus duration, 10-second interstimulus interval; stimuli increased in 50-pA steps over 20 sweeps, resulting in evoked current recordings from 50 to 1,000 pA). Electrophysiological data were analyzed using Clampfit software.

Only one cell was recorded per dish. After recording, the cell's location was marked by physically noting it on the underside of the plastic culture dish and capturing a digital image using a Scientifica monochrome camera and SCIght 2.0 software. The bath solution was then replaced with 4% PFA in PBS for 10 min, followed by rinsing and replacing with 100% PBS solution. Cells were stored at 4°C until immunolabeling procedures were carried out. Immunocytochemistry was performed to examine Atf3 expression in recorded DRG neurons.

### Bioinformatics

Putative *ATF3*-regulated human genes were identified from the GSEA website (https://www.gsea-msigdb.org/) using data from TRANSFAC (v7.4). These were cross-referenced against genes assigned with ontology annotations (from AmiGO): “regulation of membrane potential” (biological process, GO:0042391), “response to pain” (biological process, GO:0048266), and/or “sensory perception of pain” (biological process, GO:0019233).

### Statistical analyses

Statistical analyses were performed using SigmaPlot/SigmaStat (Systat Software, San Jose, CA, USA). First-pass analysis to examine differences between the skin-incised and control groups for all assessments was done using analysis of variance (ANOVA) or repeated measures ANOVA and was followed by pair-wise comparisons (Student-Newman-Keuls). Differences were considered to be statistically significant if *p* < 0.05. Data is presented as mean ± standard deviation.

### Terminology

In this report we use terms that are often considered interchangeable, but we make distinctions which we believe are, or will be, meaningful.

Injury vs. Damage: We use “injury” to imply the action, and “damage” to imply the result/condition.

Tissue damage vs. Incision: We are considering “incision” as one means, among many, of inducing “tissue damage”. “Incision” refers to clinical surgical practice and the current model and data, while “tissue damage” refers to the broader meaning and impact.

Nerve injury and axon injury: We consider these from the perspective of Gross Anatomy and Histology, where these are considered related but distinct due to different features, predominantly of the cellular complement. “Nerve injury” refers to injury of the Gross Anatomical structure of a peripheral nerve, whereas “axon injury” refers to injury to axons irrespective of the tissue in which they are resident. Further details are provided in Discussion.

## Results

### Nerve conduction in the acute phase of tissue damage is not required for *Atf3* upregulation

Local and regional anesthesia is routine and indispensable for many surgical procedures and to offer temporary relief for painful conditions (34). Although this approach largely provides excellent relief/prevention of pain in the short-term, preventing the emergence of longer-term pain has had mixed results (34). If the induction of an injury-like/cell-stress-like response in sensory neurons – indicated by expression of *Atf3* – is part of the mechanism of persistent pain, then one would expect that *Atf3* expression might occur irrespective of the use of conduction-blocking drugs.

**Figure 1:**
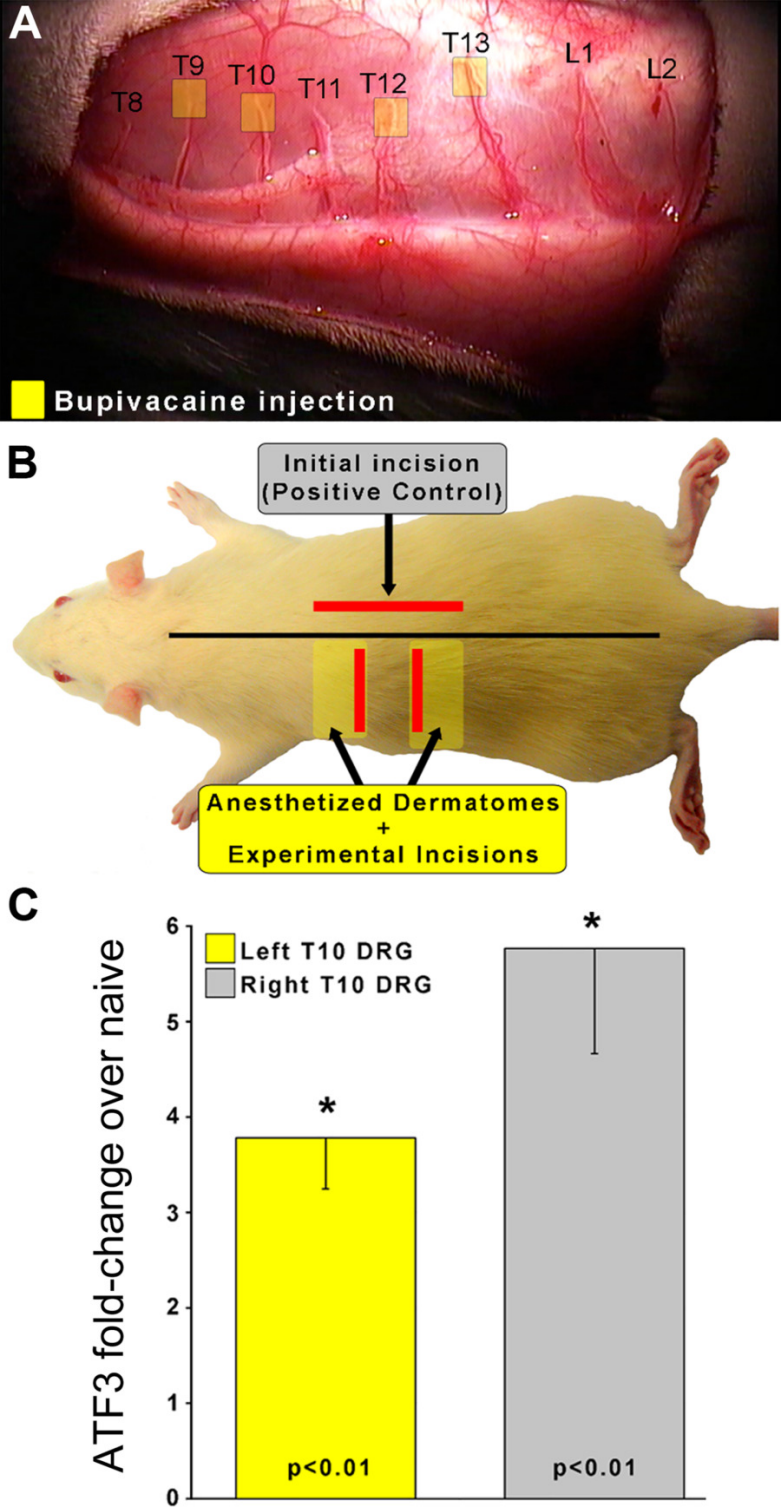
To determine if nerve conduction is required for *Atf3* expression, a skin incision was made after injection of local anesthetic to the left-side T9, 10, 12, and 13 Dorsal Cutaneous nerves (yellow boxes in panel **A**). This resulted in insensate skin regions (yellow boxes in panel **B**). Incisions (red lines in panel **B**) were made in skin that was fully sensate (right-side of animal; positive control without local anesthetic to nerves) or into insensate skin (left side of animal). *Atf3* mRNA expression is indicated in panel **C**. *P*-values provided are for *post-hoc t*-test vs. naive.

We sought to determine if clinically-modeled treatment with local anesthetics could prevent induction of *Atf3*. As indicated in Figure 1 we performed a longitudinal incision to the right of midline, beyond the reach of any left-side cross-midline innervation. This initial incision serves both to expose the left-side dorsal cutaneous nerves for accurate peri-nerve administration of the anesthetics, but also served as the positive control incision affecting many segments on the right side of the animal. We then applied a single bolus of bupivacaine (0.5%, clinical formulation) to create a fascia-contained dome of fluid surrounding a 3–5 mm portion of each of the T9, 10, 12, and 13 dorsal cutaneous nerves.

We then identified the regions of skin that were insensate by using light pinch or prick with the sharp tips of #5 forceps and observing for the Cutaneous Trunci Muscle Reflex (CTMR) (35–39). The CTMR is resistant to the pentobarbital anesthesia used for surgical procedures with these animals. The regions of insensate skin were marked and incisions made inside those insensate regions (Figure 1). Incisions were made toward the edge of the insensate zones to avoid cutting the dorsal cutaneous nerve trunks themselves as they penetrated the skin.

To address the unlikely-but-possible scenario that this approach might block conduction by fluid compressing the nerve (and thus potentially inducing Atf3 expression on its own) we performed a similar application of saline around unused dorsal cutaneous nerves. The CTMR could still be driven by stimuli applied to skin regions innervated by those nerves, indicating continuity of signal conduction.

As expected, *Atf3* expression in the right-side DRG samples was strongly upregulated. It was also clear that clinically-modelled use of local anesthetic did not prevent incision-induced expression of *Atf3*, as the expression in left-side DRG samples was also strongly upregulated.

It is possible that the anesthetic may have modulated the *Atf3* response to some degree, but making such a comparison in this surgical model is not entirely appropriate as the incisions on the left and right side were different and likely affected different numbers of sensory neurons. The functional control we performed strongly indicated that the peri-nerve bolus application of anesthetic did not compress the nerve. This is further suggested by the similarity of *Atf3* expression between the positive control (skin incision) and the experimental (incision + anesthetic) conditions. If the injection had compressed the nerve the magnitude of *Atf3* expression may have been dramatically greater.

Clinical relevance: Bupivacaine (as well as other anesthetics) is commonly used as a nerve block given prior to surgery to prevent nociception. Despite incision into skin made insensate by successful nerve block, *Atf3* is still upregulated.

### Axonal damage is required for *Atf3* upregulation

**Figure 2:**
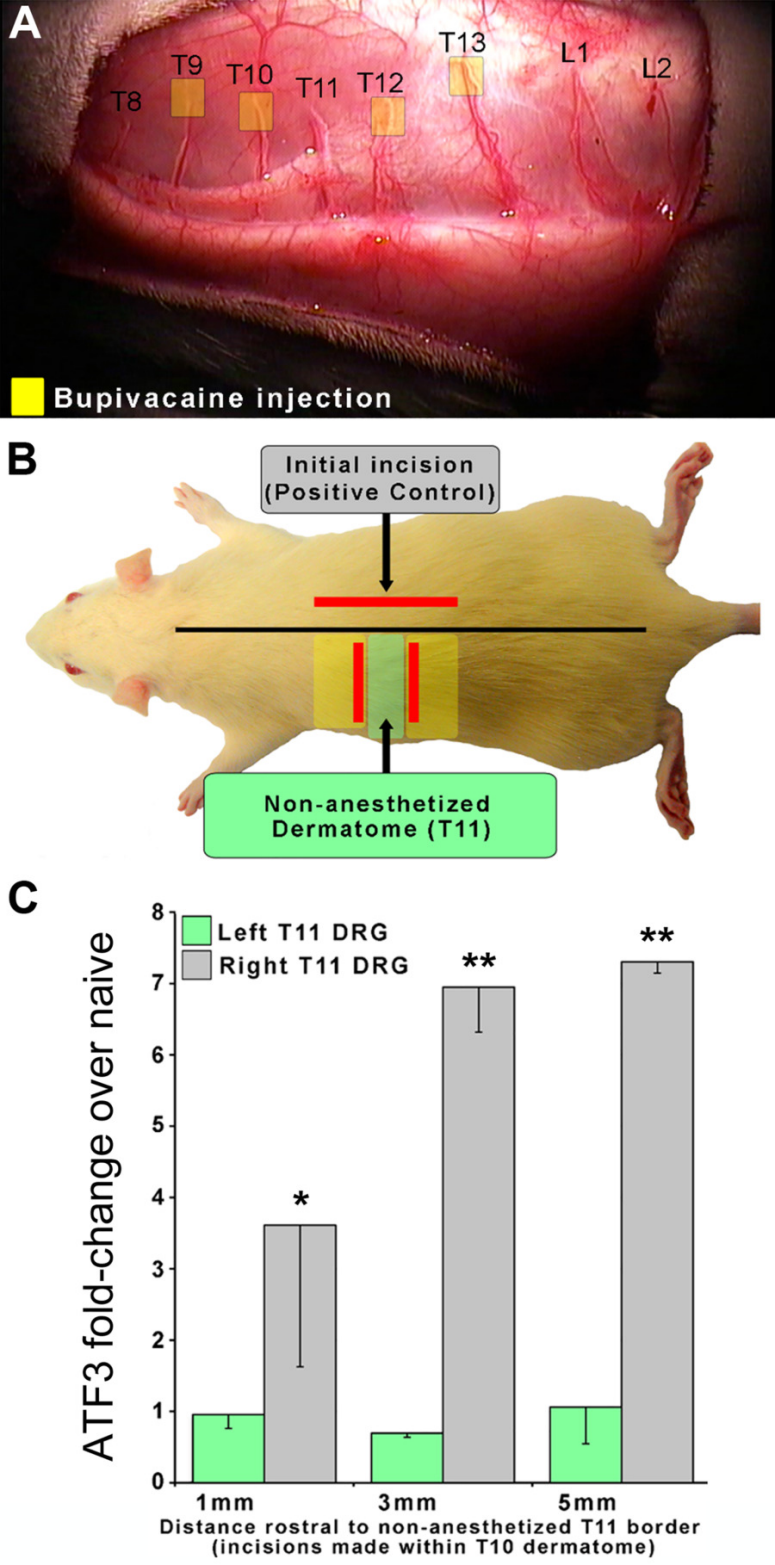
To determine if axonal damage is necessary for *Atf3* expression, an experimental design similar to that described for Figure 1 was used. In this case, local anesthetic to the left-side T9, 10, 12, and 13 Dorsal Cutaneous nerves (yellow boxes in panel **A**) was used to reveal the full extent of the T11 dermatome so that we could ensure that incisions were placed outside and did not damage T11 axons. Incisions (red lines in panel **B**) were made 1, 3, or 5 mm outside of the non-anesthetized dermatome (T11). *Atf3* mRNA expression is shown in panel **C**. *P*-values are for *post-hoc t*-test vs. naive.

In order to assess the requirement for axonal injury, as opposed to just an inflammatory environment, we examined whether inflammation alone could induce *Atf3* expression in DRG. We anesthetized the T9, 10, 12, 13 dorsal cutaneous nerves to allow us to define the T11 dorsal cutaneous nerve receptive field using the CTMR. Once spatially defined, we created a full-thickness incision that followed the (generally linear) border of the T11 dermatome. For these incisions, we borrowed from previous findings (39) which indicated that inflammation-mediating proteins were found in high concentration in the skin up to at least 1 mm away from an incision, but were significantly lower (though still increased) at 2 mm. Working from their data, our design included a group that should have had axons from the Left T11 DRG exposed to levels of inflammatory mediators varying from high (1 mm) to low/nil (5 mm). If inflammation alone were sufficient to induce *Atf3* expression in sensory neurons, we would expect to see *Atf3* expressed in the DRGs from animals with incisions 1 mm outside the T11 dermatome but not from those with incisions 5 mm outside the T11 dermatome. Instead, we found that none of the left-side DRGs expressed Atf3 above naive levels, indicating that presence of inflammatory mediators alone is not sufficient to induce expression of *Atf3* (Figure 2). This indicates that actual damage of axons innervating the injured tissue is necessary for induction of *Atf3* expression in the sensory neurons.

Clinical relevance: Tissue reaction processes (including inflammation) in the region of overt tissue damage (as modeled here) are not sufficient to induce the injury/stress gene regulation response in sensory neurons innervating the tissue.

### Suppressing inflammation does not prevent *Atf3* upregulation

It appears that inflammation is likely not sufficient to induce *Atf3* expression, at least as induced here and at the time point we assessed. To determine the necessity of inflammation for tissue damage-induced *Atf3* expression, we examined whether administration of an anti-inflammatory drug (ketoprofen) (40) might regulate *Atf3* expression after incision. We further assessed whether a combination of ketoprofen and local anesthetic (lidocaine) administration – a common clinical pain-control regimen – could prevent *Atf3* expression.

**Figure 3:**
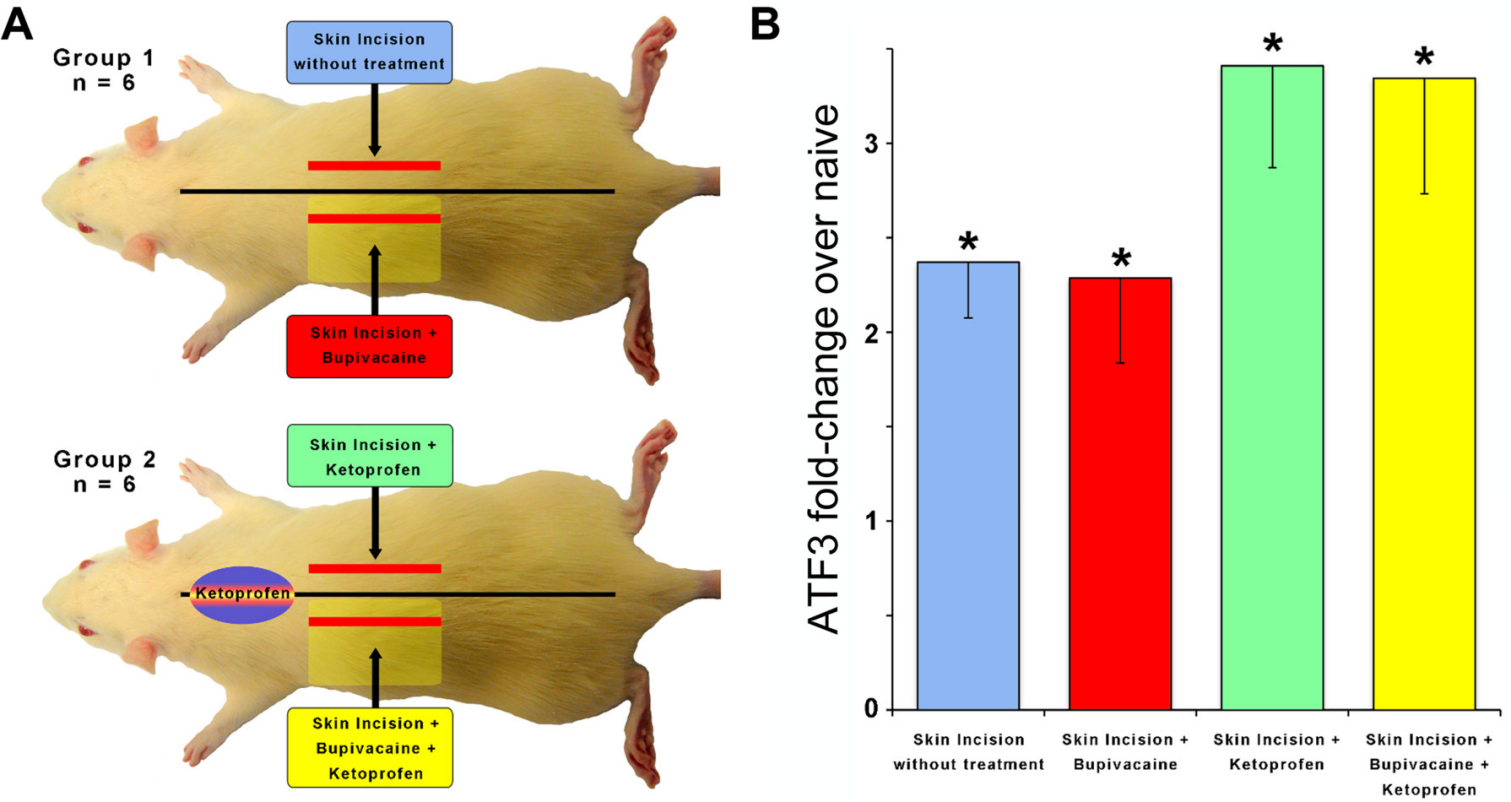
To determine if inflammation is required for *Atf3* expression, a skin incision was made after injection of an anti-inflammatory (Ketoprofen; 10 mg/kg)(Panel **A**). Incision with/without local anesthetic (Bupivacaine; 0.125% in dH20 to dorsal cutaneous nerves serving the incised skin) was also tested (Panel **A**). *Atf3* mRNA expression is shown in panel **B**. *P*-values are for *post-hoc t*-test vs. naive.

The incisions for these experiments were longitudinal – parallel to the midline – so we included additional animals with similar treatments as reported in Figure 1 to provide a more accurate comparison (Group 1). The induction of *Atf3* expression in DRG innervating incised skin was not prevented or reduced by anti-inflammatory treatment (Figure 3, green). Induction of *Atf3* was also not prevented or reduced by combined treatment with an anti-inflammatory and local anesthetic (Figure 3, yellow). These data suggest that inflammation is also not necessary for induction of *Atf3* in sensory neurons innervating damaged skin.

Clinical relevance: Ketoprofen is used peri-surgically to suppress the inflammatory response and reduce surgical pain. Despite the administration of Ketoprofen, or both Ketoprofen and Bupivacaine, *Atf3* expression is still induced in DRG housing sensory neurons innervating the incised skin.

### Repeated tissue damage extends mechanical hypersensitivity in mice

**Figure 4:**
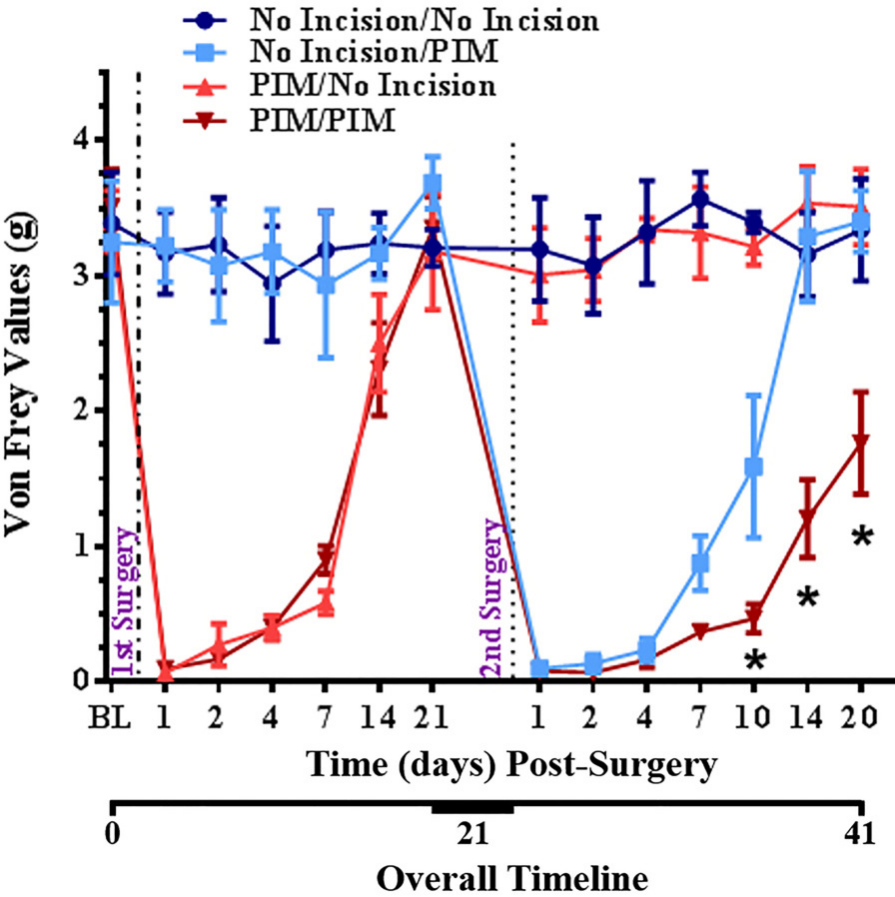
Changes in mechanical threshold after no incision, single incision on Day 0, single incision on Day 0, single incision after 3 weeks, or repeated incision on Day 0 and 3 weeks in mice. Surgeries are indicated by vertical dotted lines. BL = pre-surgical baseline. *p < 0.05 RM-ANOVA and *post hoc t*-test for PIM/PIM compared to No Incision/PIM group.

We examined the behavioral response to a second, repeated incision compared to a single incision in mice. We employed the well-characterized plantar incision model (PIM) of post-surgical pain (27, 28, 39, 41–52). Mechanical hypersensitivity was assessed with von Frey filaments. As expected, a single incision produced a robust mechanical hypersensitivity that peaked within a day and resolved back to pre-incision baseline within 3 weeks (Figure 4). Hypersensitivity did not develop during the same period in the No Incision (control) group. In the main experimental group (PIM/PIM), a second incision was performed as close to the same site as possible 3 weeks after the initial incision. This produced a mechanical hypersensitivity that was similar in magnitude and again peaked within a day but resolved more gradually than after the initial incision (PIM/No Incision) in this 2 × 2 experimental design. These results suggest that a history of prior incision increased the duration of incision-induced mechanical hypersensitivity.

Clinical relevance: Repeated injury is an etiological factor for persistent pain. Repeated injury in this clinically-relevant animal model induced a more robust post-operative pain-like response than single injury. Animal models of repeated tissue damage may be suitable for the identification of mechanisms underlying chronic post-surgical pain.

### Repeated tissue damage potentiates the *Atf3* response

**Figure 5:**
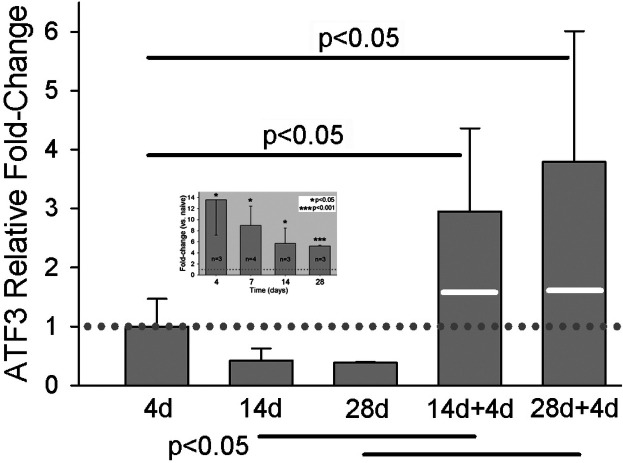
qPCR results for *Atf3* at different times after single skin incision, or two different intervals before a second skin incision. 14d + 4d indicates: 1st incision, 14 day delay, 2nd incision, 4 day delay, euthanize. Similar for 28d + 4d. Values of bars are fold-change vs. naïve, normalized against the mean of the fold-change at 4d (dotted line). White lines on the bars of the 2-incision group indicate the expression level that would be expected by simple addition of the levels from the same time points after single incisions. Data for single incision are the same as those presented in inset, which is normalized to naive. *N* = 7 for the 2-incision groups. Statistical analysis was ANOVA and *post hoc t*-test. *P*-values in the figure are for the comparisons indicated by the solid horizonal bars.

Single incision results in a significant increase in *Atf3* mRNA and protein expression in DRG innervating incised skin (10). This increase abates somewhat over time from the initial maximum but remains a highly significant increase even out to 28 days post-incision (Figure 5 inset).

Because one of the greatest predictors of chronic pain is repeated injury (53), we examined the effect of repeated incision on expression of *Atf3* in DRG that innervate the injury site. We hypothesized skin incision might “prime” *Atf3*-expression to subsequent incision, as observed after repeated nerve injury (54, 55). We induced a second incision, as close to the original incision as possible, at either 14 or 28 days after the original incision. We then took tissue 4 days later, at the peak of single-incision *Atf3* expression. As illustrated in Figure 5, the second incision induced a dramatic increase in Atf3. These data indicate that repeated tissue injury may indeed induce a priming/conditioning response for *Atf3* expression.

**Figure 6:**
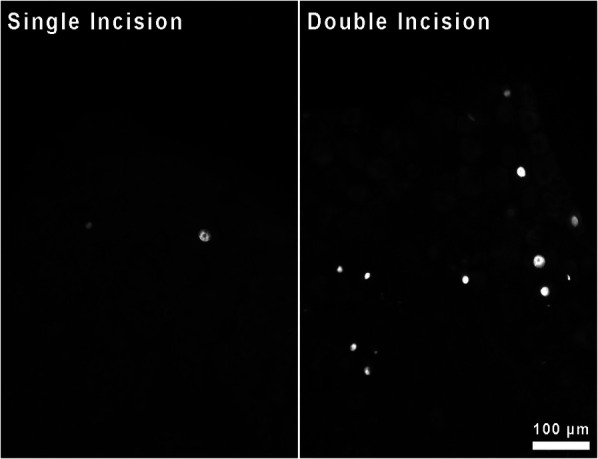
Immunohistochemical staining for Atf3 protein in sections of DRG housing neurons innervating skin incised either once or twice.

Qualitatively, it appears that the increased expression of *Atf3* at the mRNA level is attributable to neuronal expression of *Atf3* (Figure 6). Much like with single incision, we did not observe *Atf3* immunohistochemical signal outside of neuron-like profiles. The increased *Atf3* signal likely includes expression by more neurons, but certainly could include more *Atf3* expression per neuron as well. We made no effort in these assessments to quantify either neuron number or degree of protein expression overall or per cell.

Clinical relevance: Repeated injury to the same region of tissue results in a robust induction of *Atf3* expression in sensory neurons that innervate injured tissue.

### Bioinformatic analysis identifies pain-related genes with *ATF3* binding-sites

Nerve injury alters expression of ion channels, some of which are associated with pain. An extensive search of PubMed revealed no empirical evidence for ATF3 directly regulating expression of any ion channels in any setting. Although there is no direct evidence, it is clear that expression of both ATF3 and some ion channels is regulated in some of the same conditions. In order to focus our search for relevant genes, we sought to determine if any known pain-related genes had structures suggesting that ATF3 may play a role in regulating their expression. We examined the Molecular Signatures Database (MSigDB; Broad Institute; Human Motif gene sets – transcription factor targets) populated by the TRANSFAC data (v7.4). We examined the human genes having at least one occurrence of the following highly conserved motifs in the regions spanning 4 kb centered on their transcription starting sites [−2 kb, +2 kb]: CBCTGACGTCANCS = 257 genes, TGACGTCA = 235 genes, TGAYRTCA = 551 genes. The total number of genes with promoters containing at least one of these ATF3 consensus binding motifs was 671. We combined the results of three gene sets (1 for each of 3 separate ATF3 binding sequences) and cross-referenced this list against a set of Gene Ontology terms related to nociception or pain and against PubMed terms for pain. Of the genes with an ATF3-binding sequence, only 18 also had pain-related annotations (Table 2).

**Table 2:** Intersectional results of genes with both Atf3-binding sites and GO term annotations relevant to pain.

Atf3-Binding Sites		Relevant GO annotations		
Genes	# sites	Regulation of membrane potential	Response to pain	Sensory perception of pain
ADAM11	2		YES	
CALCA	1		YES	YES
BNIP3l	3	YES		
CACNA1G	2	YES		
CALM1	1	YES		
CAMK2D	2	YES		
CFTR	1	YES		
FGF12	1	YES		
GRIA4	1	YES		
GRIN1	1	YES		
HCN4	1	YES		
KCNA5	2	YES		
KCNF1	2	YES		
KCNN2	2	YES		
P2RX3	1	YES		
PHOX2B	2	YES		
PLN	1	YES		
SCN3B	2	YES		

The full list of results is available in Supplementary Data.

### Pain-related genes with *ATF3* binding-sites demonstrate unique expression after repeated incision

Skin incision is associated with significant electrophysiological changes in the Atf3-expressing sensory neurons (10), leading us to prioritize consideration of the 6 genes for voltage-gated ion channels. We chose to examine the expression of two genes known to influence electrical signaling in sensory neurons, particularly the depolarization phase of the action potential – *Scn3b* (Voltage-gated sodium channel beta subunit 3) (https://www.ncbi.nlm.nih.gov/gene/55800) and *Cacna1g* (*Cav3.1*/T-type low-voltage-activated calcium channel) (https://www.ncbi.nlm.nih.gov/gene/8913).

**Figure 7:**
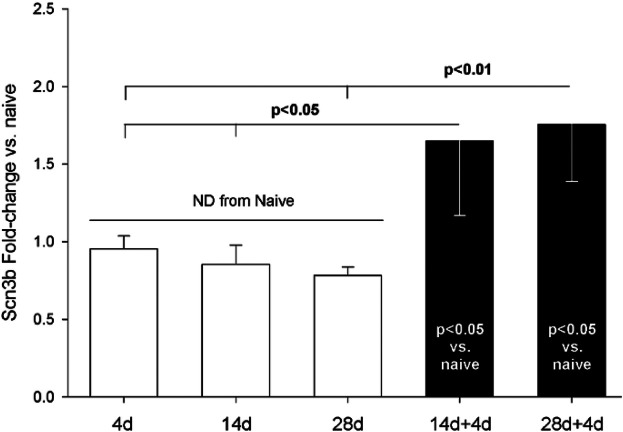
qPCR assessment of Scn3b mRNA from DRG housing sensory neurons innervating skin incised once (white bars) or twice (black bars). Single incisions were 4, 14, or 28 days earlier. Naïve (*n* = 4), 4d (4), 14d (4), 28d (4), 14 + 4 (7), 28 + 4 (7). Statistical test was ANOVA and *post hoc t*-test. *P*-values are from *t*-test. Scn3b expression was normalized to GAPDH. All groups were normalized to mean of Scn3b-v-Naïve, which was set to “1”.

*Scn3b* mRNA expression appeared to be unaffected during the 4 weeks following the first incision (Figure 7, white bars). However, *Scn3b* expression was significantly increased after the second incision (Figure 7, black bars).

**Figure 8:**
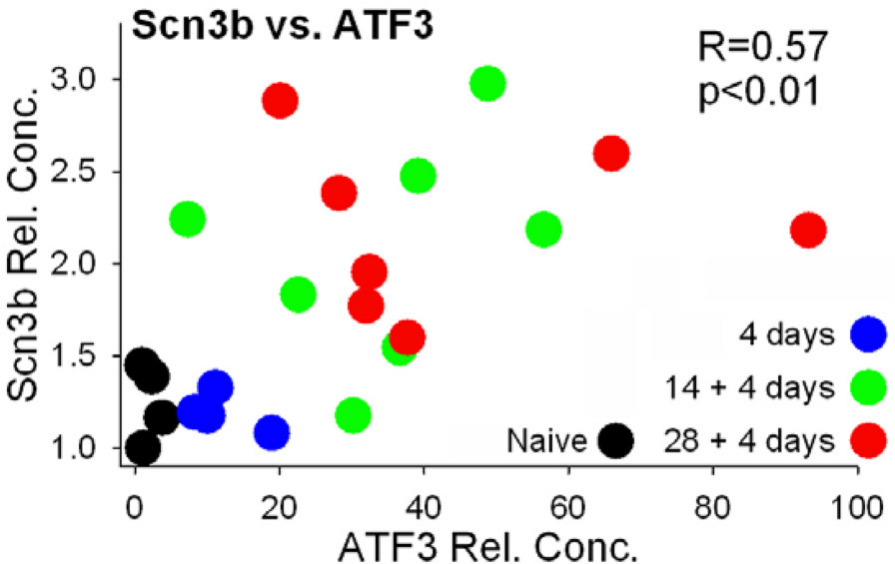
Expression values of Scn3b and Atf3 (both normalized to GAPDH). Values were compared by Pearson correlation.

*Scn3b* was identified by screening genes with *Atf3*-binding sites for pain-related annotations. We therefore examined whether there may be a relationship between expression of *Atf3* and *Scn3b* on an animal-by-animal basis across all groups. There was a significant positive relationship between expression of *Atf3* and *Scn3b* (each vs. GAPDH) (Figure 8).

**Figure 9:**
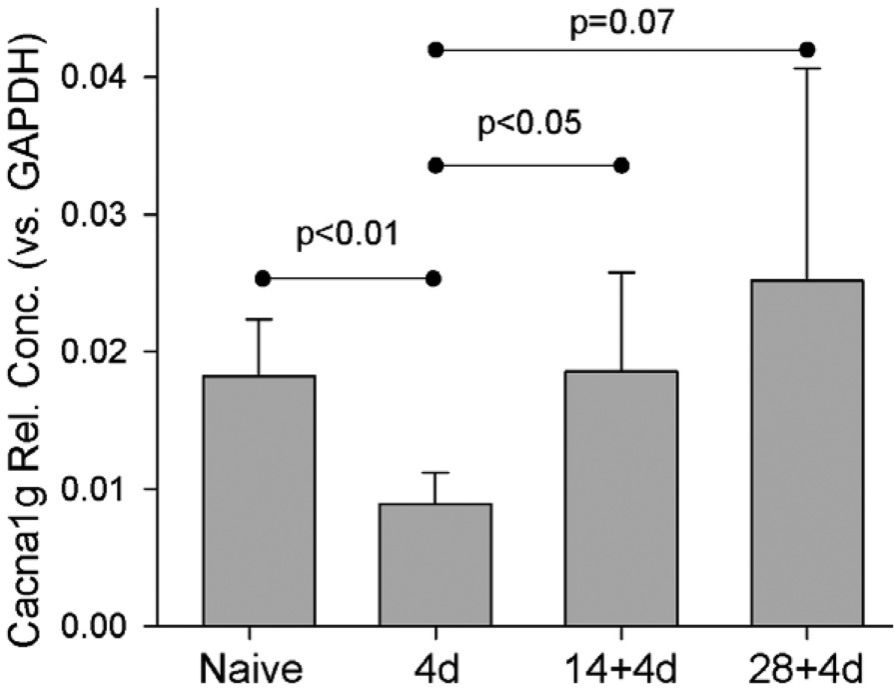
Naïve (*n* = 4), 4d (4), 14d (4), 28d (4), 14 + 4 (7), 28 + 4 (7). Statistical test was ANOVA and *post hoc t*-test. *P*-values are from *t*-test. Cacna1g expression was normalized to GAPDH.

Unlike *Scn3b* mRNA, expression of *Cacna1g* mRNA was significantly reduced by 4 days after a single skin incision (Figure 9). This significantly-reduced expression was reversed 4 days after a second incision. Expression after repeated incision was no different from naïve, while after single incision it was significantly reduced.

**Figure 10:**
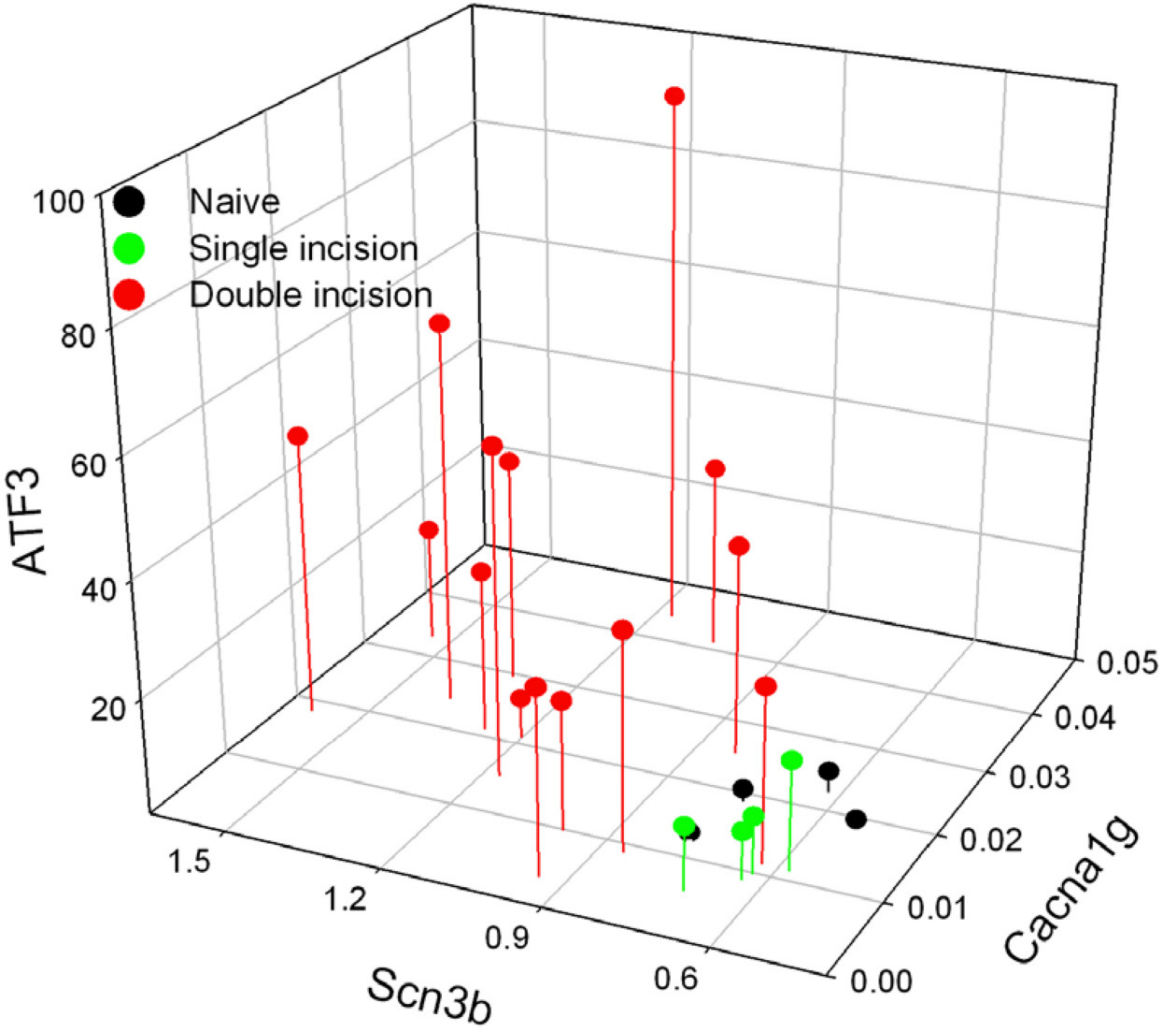
The relative concentration (vs. GAPDH) of each of the genes is plotted on a per-animal basis. Both groups of animals with two incisions (14 + 4 and 28 + 4) are labelled as Double incision.

We then compared the levels of each of the genes within each animal. Because expression of each gene was different between conditions of single- and repeated-injury but was similar across the 2 repeated-injury groups, we combined animals of the 14 + 4 and 28 + 4 repeated-incision groups under the same label of “Double Incision”. These are compared to those of animals of single incision and naïve groups (Figure 10). It is clear that the expression from naïve and single incision animals cluster together, while the expression from the double incision animals is much more distributed. Similar to the pattern of expression with *Scn3b*, expression of all 3 genes in the repeated-incision group is greatly separated from the single incision animals.

Clinical relevance: Effects on expression of pain-related genes differ between conditions of single and repeated tissue damage. These animal models suggest mechanisms by which repeated injury contributes to the etiology of, and risk for developing, persistent neuropathic pain.

### Wound closure method and treatment affect induction of *Atf3* and *Gap43* after incision in rats

Wound closure with suture or staples clearly results in changes in expression of genes in a manner similar to what occurs in nerve injury (9, 10). Glue-closure is also used with some wounds (56–62). We therefore examined the effect on gene expression of incision followed by wound-closure with surgical glue.

**Figure 11:**
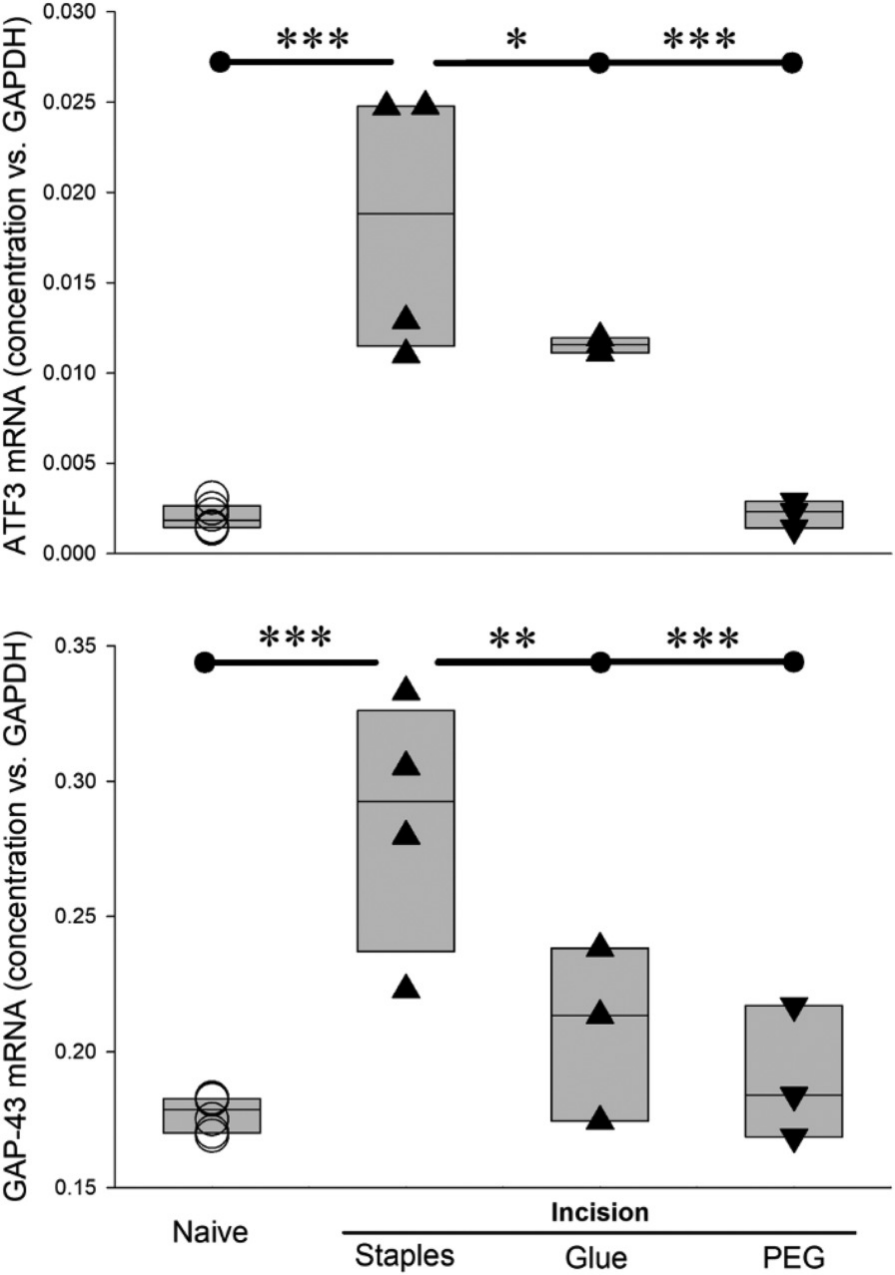
Different wound-closure methods, and post-incision treatment, affect *Atf3* and *Gap43* expression differently 4 days after incision. * = *p* < 0.05, ** = *p* < 0.01, *** = *P* < 0.001 by ANOVA and *post hoc t*-test with control set to incision + staples. Naïve (*n* = 6), Incision + staples (*n* = 4), incisio*n* + glue (*n* = 3), incision+PEG+staples (*n* = 3).

Similar to *Atf3*, *Gap43* is upregulated in sensory neurons in response to nerve injury [e.g., (55, 63, 64)] and after skin incision (9, 10). As expected, *Atf3* and *Gap43* expression was significantly increased 4 days after the incision was closed with surgical staples. Because many cutaneous wounds are closed with cyanoacrylate adhesive, we examined whether this closure method might result in a different outcome for the markers of axonal injury. These increases were significantly reduced when the wound was closed with surgical glue (Figure 11).

Our working hypothesis has been that tissue damage, including surgical incision, induces damage to axonal integrity to a sufficient degree that the positive and/or negative centripetal signals indicating injury become sufficient to induce gene expression changes similar to those induced by overt damage of nerve tissue. Under this hypothesis, we examined whether a treatment known to maintain or restore membrane integrity, including axonal membrane, might influence these changes in gene expression. Polyethylene glycol and other “fusogens” have been used for decades by labs working from this idea of membrane repair or stabilization (22, 25, 26, 65–73). The understanding of mechanisms underlying the effects of the fusogens has advanced significantly, even to the stage of enabling veterinary and human clinical trials (19, 20, 74–76).

We administered PEG acutely post-incision, applied topically to the incision site and subcutaneously as was done previously (26, 68) and closed the incision with staples. In rats treated with PEG, there was no upregulation in expression of *Atf3* or *Gap43* mRNA at 4 days after incision (Figure 11), a time chosen because it appears to be the peak of the *Atf3* response.

**Figure 12:**
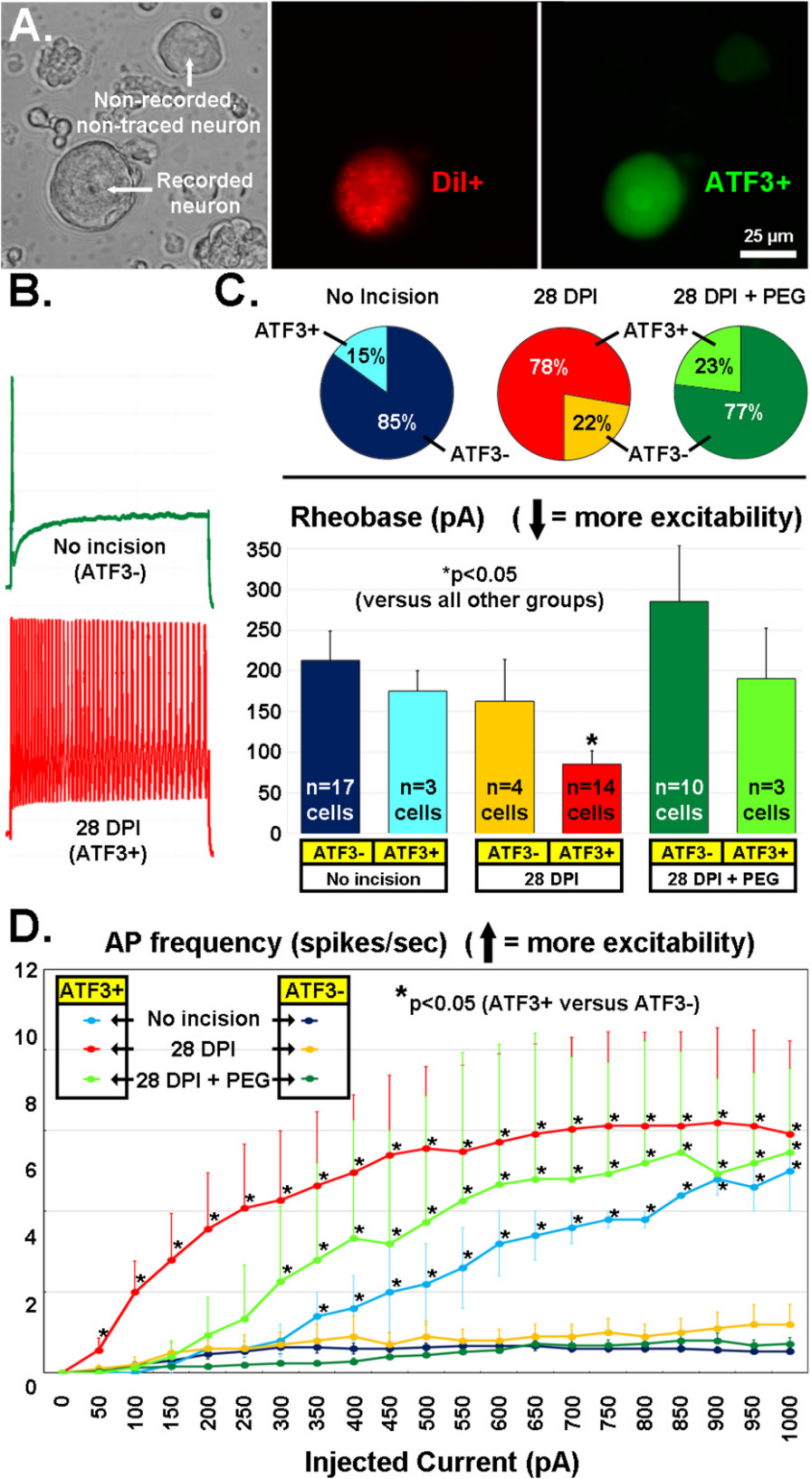
**(A)** Recordings were made of acutely-dissociated DRG neurons (left panel, DIC image) labeled from the incision site with the retrograde tracer DiI (middle panel, red) and later run for Atf3 immunohistochemistry (right panel, green). **(B)** Examples of firing pattern (in response to a 1s, 1000pA depolarization) from an Atf3^−^ neuron from a naïve animal (green) and from an Atf3^+^ neuron traced from the wound site 28d after incision. **(C)** Proportion of DiI-traced and recorded neurons that expressed Atf3 in the 3 different treatment groups (pie-charts). Rheobase current of DiI-traced and recorded neurons parsed by expression of Atf3 in the 3 different treatment groups (bar graphs). **(D)** Number of APs fired (*y*-axis) during a 1s depolarizing pulse (*x*-axis). Error bars are SD. * = *p* < 0.05 in **(C)** (vs. all other groups) and **(D)** (Atf3^+^ vs. Atf3^−^).

Following the promising early-stage results from PEG-treatment on expression of *Atf3* and *Gap43* mRNA, we performed additional experiments to examine expression of *Atf3* protein and functional properties of sensory neurons traced from the incision site, similar to our prior work (9, 10, 77–32). We assessed some of the excitability-related electrophysiological properties of sensory neurons DiI-traced from the incision site or an equivalent site in naïve non-incised animals using patch clamp of single-neurons dissociated 28 days after incision. Following recordings, the cells were fixed and assessed with immunohistochemistry against *Atf3*. This provided a means to directly associate the *Atf3* expression with electrophysiological properties at the single neuron level (Figure 12A).

Rheobase (depolarizing current threshold for inducing action potential firing; Figure 12B, green trace; 12C bar graphs) was significantly lower in Atf3^+^ vs. Atf3^−^ in the incision group, and was the lowest (most-excitable) vs. all other groups, in agreement with our prior work (10). Also as expected, the proportion of traced neurons that were Atf3^+^ was greater in the incision group (Figure 12C, red/yellow pie charts). Interestingly, the proportion of Atf3^+^ neurons was reduced in the PEG-treated group (Figure 12D, green pie charts), similar to the proportion from the traced/non-incised group (Figure 12D, blue pie charts).

As we reported previously (10), many of the Atf3^+^ neurons displayed repetitive firing in response to even low-level depolarizing currents (Figure 12B, red trace), which does not usually occur with acutely-dissociated sensory neurons. We quantified this response profile and determined that the Atf3^+^ neurons displayed significantly greater repetitive firing than Atf3^−^ neurons (which had essentially no repetitive firing), regardless of group (Figure 12D). This significant difference occurred at much lower levels of depolarizing stimulation for the incision group than for the tracer-only groups and the incision+PEG+staples group.

Clinical relevance: Acute-stage application of PEG may prevent long-term injury-related changes in gene expression and electrophysiological properties, which might in-turn reduce persistent pain after tissue damage. There is also reason to suspect that glue-closure may provide better pain-outcomes than suture or staples for suitable conditions.

## Discussion

Everyone experiences pain after severe tissue damage or surgery. This can persist beyond apparent wound healing, present as resistant to anti-inflammatory treatments, and include neuropathic components. Current management of post-surgical pain is imperfect, and both research and clinical practice are calling for novel perspectives, inclusion of more mechanism-based diagnoses, and more effective treatments (6, 81, 82). Here we consider the contribution of axonal damage as a neuropathic factor. To address this question, we: (1) administered conventional analgesic drugs; (2) examined the effects of repeated injury on pain hypersensitivity and axonal damage; and (3) examined the efficacy of novel approaches to prevent the nerve injury-like response in sensory neurons.

We used the *de novo* expression of *Atf3* mRNA as a surrogate for the larger functional effects that we described previously (10). Pre-surgical local/regional anesthesia and perioperative administration of anti-inflammatory drugs can reduce post-surgical pain, but do not prevent persistent pain (6, 83). If *Atf3* expression were to be reduced/blocked by local anesthesia or anti-inflammatory treatment, then it would not be a feasible potential mechanism for persistent pain. Instead, we found that the anti-inflammatory drug ketoprofen did not change *Atf3* expression (Figure 3). Similarly, local anesthetic did not change *Atf3* expression, indicating both that AP conduction from incision site to DRG is not required for the response and suggesting that standard clinical local/regional anesthetic approaches are not likely to affect this response (Figure 1). These findings alone provide a strong validation for this response as a potential contributor to clinical persistent post-surgical pain as the response appears resistant to the most-used pain-control approaches.

Although we did not set out to test the threshold for what stimuli are required to induce *Atf3* expression in sensory neurons, the study nonetheless offers some insight. The dermatome-mapping process itself – inducing the CTM reflex by noxious pinch – was not sufficient to induce detectable *Atf3* expression (Figures 1–3). For example, the mapping process for Figure 2 activated signaling in T11 axons without induction of *Atf3* in the T11 DRG. This is consistent with our previous study showing that noxious pinch did not induce *Atf3* expression (84).

### Considering axonal injury in skin vs. nerve

Our data promote the idea that post-surgical pain has a neuropathic component which is resident in sensory neurons. Although we show that some aspects of the sensory neuron response to injury of a nerve are evident in sensory neurons after skin incision, it is not yet clear that all aspects of the response are the same. Determining whether the sensory neuron response to damage of their axons in nerve tissue is essentially the same as, or meaningfully different from, damage of their axons in target tissue is vital for understanding mechanisms of tissue damage-induced pain, including post-surgical. In this context we should consider how the core injury – that of injury to the axon – might differ in the setting of skin vs. nerve. The full neural “cell body response” to nerve injury requires both positive/injury signals (such as gp130 cytokines) and negative/absence signals (e.g., lack of constitutive retrogradely-transported target-derived factors such as NGF/TrkA or NT-3/TrkC) (85–89). Injury to a discrete peripheral nerve induces positive/injury signals directly in the injured nerve tissue which signal locally and by retrograde signaling to the soma/nucleus of the injured neurons. Nerve injury also results in negative/absence signals by virtue of disconnecting the biochemical transport pathway from target tissue to soma. Injury to target tissues (such as in our model) also induces positive injury signals that are presumably retrogradely transported similar to what occurs with nerve injury, though it is not yet known if those signals are the same or different from the injury signals induced by injury to nerve tissue. It is also possible that because the injured axons are still resident in their target tissue there may not be a negative/absence signal (i.e., the axons may still have access to those target-derived signals and retrogradely transport them). That is, target-derived positive/intact signaling – electrical and/or biochemical – may remain at a level that is sufficient to either entirely or partially prevent the positive/injury signal from inducing a cell body response. It is this possibility that was tested by the incisions made at progressive distances outside of a defined receptive field/dermatome (Figure 2). The conservative perspective was to hypothesize that the positive injury/inflammation signals induced by the incision 1 mm outside the dermatome would be capable of inducing *Atf3*, and the incision at 5 mm outside would not. But this did not occur for any distance, suggesting that the level/type of positive tissue-injury/inflammation signal induced by incision used here was not capable of inducing *Atf3*. This clearly implies that overt injury to the tissue-resident axons is required for induction of *Atf3* expression.

Although we show here that inflammation did not induce a nerve injury-like response (i.e., *Atf3* expression), inflammation of peripheral target tissues can induce many of the same effects we have described – i.e., sensitization of electrophysiological properties/responses and nociceptive behavioral responses. However, those are largely prevented by anti-inflammatory treatments and are not neuropathic. Nonetheless, it is possible that severe instances of inflammation and chemical stimuli which may themselves result in damage to the tissue or the responsive axons are capable of inducing *Atf3* expression in the sensory neurons innervating the inflamed tissue. This has been demonstrated experimentally, though it is possible that some of the stimuli were non-physiologic (90).

The distinction we consider here between axonal injury associated with target tissue damage and nerve damage may appear esoteric, but it is not. First, it has been recognized for over 100 years that injury to dorsal root and to peripheral nerve result in different sensory neuron responses, with more recent studies providing some molecular and cellular signatures (54, 91–93). It may be that there is yet another different response when axons are injured within the physical setting of their target tissue with a cellular composition distinct from that of nerve tissue. Single axons of sensory neurons traverse many different tissues, each of which has a distinct cellular composition which may become altered differently by injury.

Second, Schwann cells are intimately associated with peripheral nervous system (PNS) axons across all of the tissues through which nerves (and axons) traverse. It is increasingly recognized that there is a range of Schwann cell types which differ in accord with the axonal composition of a nerve (sensory or motor) and their central/peripheral and tissue location (94–96). Terminal Schwann cells differ with the tissue/structure innervated (97, 98). Axonal injury induces differentiation of a “Repair Schwann cell” phenotype (99–102). It is entirely feasible that the interaction between damaged axons and their associated Schwann cells may therefore differ by location of injury – e.g., in nerve tissue or in target tissue.

Third, outside of an overt injury to a distinct peripheral nerve, there is little recognition of neuropathic mechanisms when considering potential factors contributing to tissue damage- or surgery-associated persistent pain. If neuropathic mechanisms are considered outside of overt nerve injury, these are often characterized as idiopathic or covert injuries to nerves, largely as a diagnosis of exclusion when anti-inflammatory treatments fail. In these cases, the field looks to any number of central nervous system (CNS) mechanisms. However, it is possible that tissue damage from any range of sources results in concurrent damage not to discrete peripheral nerves, but to tissue-intrinsic axons, and that this axonal damage triggers a cellular injury response in sensory neurons that resembles the response induced by nerve injury. Such a neuropathic mechanism would require therapeutic approaches appropriate for the PNS, which often differ from those required for CNS pathologies.

### Considering repeated damage

It is uncommon that a single incident of tissue damage, surgical or otherwise, results in persistent neuropathic pain. Anti-inflammatory drugs are usually highly-effective for pain-control in these cases. Nonetheless, this can and does occur for conditions such as Complex Regional Pain Syndrome. It is becoming increasingly recognized that labelling the precipitating event as a “single non-notable injury” may be misleading and incomplete, as there may be predisposing factors that are not readily apparent in the patient's medical history (103–105). It is a fundamental principle of biology that prior experience can influence the response to later similar experience. Relevant here is that there is suggestion from both clinical experience and basic science that repeated injury can change and/or amplify the response, including from the most common surgery performed globally – Cesarean section – from which chronic post-surgical pain is not rare [e.g., (54, 89, 106–117)].

We therefore examined whether the response to repeated injury might differ from single injury. The response to repeated injury assessed behaviorally indicated a longer-lasting hyperalgesia response than for single incision. It remains possible that the second plantar incision might increase not only duration but also the degree of hyperalgesia, and that this was obscured by a floor in von Frey threshold after the first incision. Another unanswered question is exactly how long mechanical hypersensitivity persisted after the second incision. Regardless, we suggest that animal models of repeated tissue damage may be suitable for the identification of mechanisms underlying chronic post-surgical pain.

The effect of repeated injury assessed at the transcriptional level indicated unique responses. Expression of *Atf3* was increased in response to a second incision to a magnitude that is beyond what would be expected from simply adding the effects of both single incisions. It is not clear if this was due to the same cells expressing more *Atf3*, the same level of *Atf3* expressed in more cells, or both. This detail will need to be clarified as the transcription-regulation actions of *Atf3* are context-dependent based on cell type, condition, available binding partners, and the level and time course of expression [e.g., (118, 119)]. At the cellular/tissue level, it is not clear if the unique effects of repeated injury are due to an altered inflammatory response such that it is now capable of inducing *Atf3* in neurons with axons outside of the injury zone, or perhaps because the initial injury induces an increased innervation density such that a second injury affects more axons/neurons.

#### Scn3b

Although the details are not entirely clear (120), *Scn3b* can influence the current density, activation threshold, and inactivation kinetics of voltage-gated sodium channels, including those with alpha subunits Nav1.3, Nav 1.7, and Nav1.8 [e.g., (121)]. *Scn3b* is upregulated in nerve injuries and streptozotocin models of painful diabetic neuropathy (122–124), all of which are associated with expression of *Atf3* in sensory neurons (125–129). *Scn3b* is upregulated in our model uniquely after repeated injury. It is not clear if this substantially-increased expression is in the same cells expressing *Scn3b* previously (small diameter sensory neurons 122) and/or additional cells, such as the large-diameter sensory neurons that normally express only *Scn1b*.

#### Cacna1g

“T-type calcium channels are…“first responders” to depolarization. The low voltage threshold for activation of T-type channels drives their opening in response to relatively small positive changes in membrane potential” (130). T-type channels are involved in low-threshold calcium spikes, neuronal oscillations and resonance, and rebound burst firing, all of which have been associated with a range of painful conditions. Even small fluctuations in voltage mediated by these channels means that there may also be a biochemical signal because the current carries calcium, a powerful signalling molecule – “…[T-type calcium channels] contribute to regulating intracellular calcium levels near the resting potential of many cells” (130).

*Cacna1g/Cav3.1* mRNA is downregulated by single incision but returns to pre-incision levels with repeated incision. The exact nature of the unique response of *Cacna1g/Cav3.1* is not entirely clear, because we do not yet know how it is regulated at 14d and 28d after single incision. It is possible that expression continued to decline or returned to pre-incision levels. Regardless, the 14 + 4 and 28 + 4 responses differ meaningfully – the prior incision changes the response to the second incision. These changes in *Cacna1g/Cav3.1* may be even more relevant in the context of the recent discovery of incision-induced long-lasting increases in depolarizing fluctuations (11).

The trajectory of recovery from hyperalgesia after repeated incision (Figure 4) suggests it is likely to be extended in duration. It is also possible that it may not return to baseline, unlike what occurs with single incision. The Taylor lab has demonstrated that there is a latent sensitization even after single insults, the masking of which relies on opioid and NPY signaling (43, 45, 131–133). Repeated injury may also influence the masking mechanisms. It is very enticing to consider the possibility that the masking and sensory axon injury-induced sensitization mechanisms may intersect and provide a clinically-relevant mechanism for persistent pain.

Our data indicate that the regulation of *Gap43* expression in DRG neurons is similar between skin incision and nerve injury, but the regulation of *Cacna1g* and *Scn3b* are not as clearly similar between skin incision and nerve injury. It is possible that tissue injury will only partially reproduce the effects of nerve injury, and/or that repeated tissue damage may result in a response that is more similar to nerve injury than that induced by a single incidence of tissue damage. These possibilities can be readily addressed with transcriptomic approaches.

### Co-regulation with Atf3

Our additional qPCR assessments were targeted toward genes that could play a role in the long-term electrophysiological sensitization we have observed and also be regulated by *Atf3*. We therefore considered whether there might be signs of co-regulation. *Atf3* and *Scn3b* were similarly-regulated, and the expression levels for DRG from repeated injury grouped well outside of the expression levels from single injury (Figure 10). *Cacna1g* displayed fairly variable expression yet still contributed strongly to a picture of consistent co-regulation of both ion channel genes with *Atf3* at the level of the individual animal. Determining whether *Atf3* directly regulates expression of *Scn3b*, *Cacna1g*, and the other genes in Table 2 could be very informative for understanding mechanisms. This would require approaches such as performing similar assessments using *Atf3*-null mice.

An additional consideration is that splice isoforms have been described for *Cacna1g*, *Scn3b*, and *Atf3* (134–136). This means that the qPCR signal we report here might be reflective of expression of different isoforms instead of, or in addition to, regulation of the number of copies. Alternative splicing can introduce even greater functional plasticity than up- or down-regulation of a single type of transcript (137). *Cacna1g* meets all of the rationally-constructed criteria for having functionally significant splice variants (138), and isoforms of *Atf3* have been experimentally demonstrated to be expressed and exert different effects on transcription (135).

### Considering the response among other known processes and factors

The biological responses described here could provide mechanisms contributing to currently unknown and/or poorly understood etiological factors for persistent pain after tissue damage. It is clear that not all incisions or tissue damage incidents are chronically-painful or sensitized, despite the responses we present here appearing to be quite powerful and consistent. Clearly the responses we describe here do not alone dictate outcomes but are instead working in conjunction with other responses and systems.

If a controlling factor in the emergence of persistent pain after tissue damage is the total number of neurons injured, then conditions with repeated injury may become more important. Neighboring non-injured axons will respond to the tissue damage-induced inflammation [which includes significantly increased production of NGF (39, 139–141)] by undergoing axonal collateral sprouting (142, 143). This could increase the number of neurons whose axons would be damaged by the second injury, ultimately increasing the number of neurons affected by the injuries.

**Figure 13:**
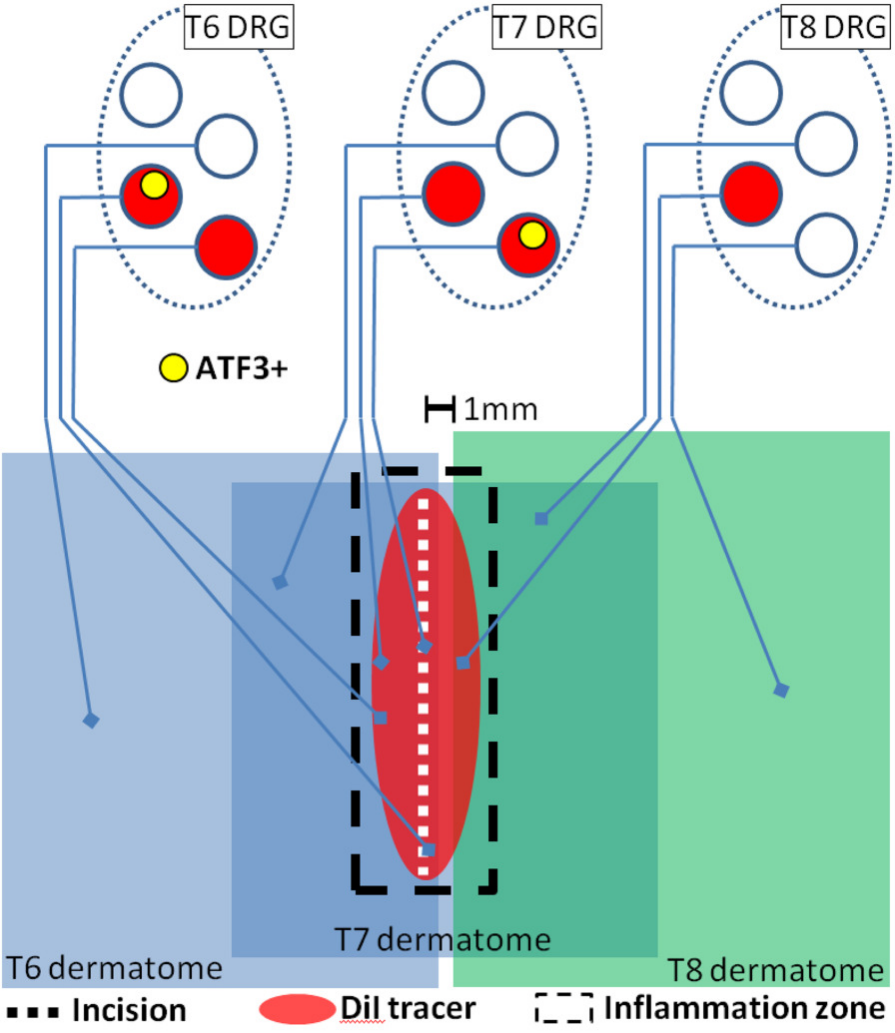
Schematic representation of presumed interaction of cutaneous neuroanatomy, retrograde tracer, and incision-associated effects. The figure incorporates conclusions from the experiments reported in Figures 2, 3, 12. Note that dermatomes are C-fiber dermatomes which overlap significantly, not the classical A-fiber dermatomes which do not overlap.

The magnitude of *Atf3* expression after skin incision was small compared to frank injury to peripheral nerve. This relatively small magnitude is essentially a function of the small proportion of neurons in each DRG that is involved in the tissue damage (Figure 13) (9, 10). Just a few sensory neurons which express Atf3 could still be a controlling factor in the emergence of neuropathic pain (144–146). It is currently unclear, but it is possible that the injury response in sensory neurons induced by tissue damage might induce similar responses in spinal cord and DRG as are induced by nerve injury such as activation of spinal microglia associated with central axons of the injured sensory neurons [e.g., (147)] and infiltration of macrophages into the DRG and localization near the soma of injured neurons where they can influence neighboring non-injured neurons [e.g., (148)]. We also cannot yet be certain that the response we describe underlies the latent sensitization described by Taylor [e.g., (43, 45, 149)], but it seems both feasible and likely that it at least contributes. We also have yet to determine how, and if, these responses interact with the range of known intrinsic analgesic adaptive mechanisms. But it is conceivable that persistent pain could emerge if the adaptive mechanisms were to become saturated (e.g., if too many sensory neurons are involved) or if some individual variance (genetic, disease, etc.) made one or more of the adaptive mechanisms less functional.

### Considerations for glue-closure

Not all wounds can be effectively and safely closed with adhesive, but glue-closure is used in many clinical settings and cyanoacrylate-type products are an integral part of the surgical arsenal. There have been many studies examining the relative benefits of sutures, staples, adhesive strips, and glues for stability, infection, cosmesis, and other factors (57–62, 150–155), but there has been little consideration of pain-outcomes (59, 150). Pain was considered in the context of closure of oral wounds (155). We have found that glue-closure obviates the nerve-injury-like changes in gene expression that are associated with suture- and staple-closure of skin incisions. The suite of data we have produced around the effects of skin incision on sensory neurons suggest that different closure methods should be considered as a factor in long-term pain outcomes.

### Considerations for PEG effects

One question is whether PEG acted to prevent or reverse expression of *Atf3*. We did not design a specific experiment to address this question. However, there are indications from separate experiments that can address this important question. The result for PEG-treatment in terms of *Atf3* mRNA expression appears inconsistent with the results for neurons expressing *Atf3* protein in the PEG-treated animals. One possibility is that the protein could have been expressed due to some injury which occurred prior to PEG treatment, and PEG did not reverse that expression. Our prior work demonstrated that a small proportion of DRG neurons are injured by the process of injecting the DiI tracer into the skin, and they express Atf3 protein even without incision (10). Indeed, the proportion of Atf3^+^ neurons remaining after PEG-treatment (Figure 12C) is similar to naive with DiI-tracing. This appears to be the most-likely scenario when the incision is separated in time from the DiI injection. It would imply that PEG-treatment is acting to prevent induction, and not to reverse the effect. If the incision were not significantly separated in time, the Atf3^+^ neurons might still have been induced prior to the PEG-treatment, but this is less likely. Thus, these combined data suggest that PEG treatment (as performed here) more likely acts to prevent induction of *Atf3* than reversing/suppressing its ongoing expression.

Taken together, PEG-treatment dramatically reduces the incision-induced and pain-associated molecular and functional indications of axonal injury and cellular stress. *Atf3* mRNA from bulk tissue is reduced, as is the number of incision-associated neurons expressing Atf3 protein. Although the electrophysiological sensitization of responsiveness of the individual Atf3^+^ neurons is still notable, *there are far fewer of those neurons remaining after PEG treatment*. Further, the PEG-treatment at the time of the incision stabilized the threshold for activation (rheobase) 28 days later, suggesting a prevention of the hypersensitivity that usually occurs after incision (for neurons expressing Atf3), as opposed to a delay.

Although PEG can have direct effects on membrane properties, a model of endothelial cell perturbation in culture suggests those effects do not endure beyond 24 h (unless PEG is continually provided) (156). This suggests that the effects on electrophysiological properties described here are likely not due to direct actions on the plasma membrane, but more likely due to persistent changes associated with the prevention of Atf3 expression.

### Treatment-mediated reduction in Atf3

The number of Atf3^+^ sensory neurons 3 weeks after induction of osteoarthritis in two separate models was reduced by treatment with an LPA receptor inhibitor (157). Since inhibitor dosing included both pre- and post- OA-induction treatment, the effect on *Atf3* expression was likely via prevention, and not reversal. Another study (158) determined that delayed administration of TLR4-antagonist could reduce the number of Atf3^+^ neurons in the relevant DRGs. It was unclear if this was due to prevention of *de novo* induction of *Atf3* in a new population of neurons in the tissue degeneration model or a reversal of expression already induced. Nonetheless, it is compelling that other treatments are also capable of reducing the overall expression of *Atf3* in sensory neurons in conditions of tissue damage.

### Study limits

Although we modeled the ketoprofen regimen from clinical use and prior experimental protocols, we did not directly characterize the nature or degree of inflammation present in this model. While we can be very confident that the ketoprofen treatment had anti-inflammatory effects, we cannot be certain of any specifics about the effects of that treatment. For example, ketoprofen reduces prostaglandin synthesis but has minimal effects on other mediators like histamines, cytokines, bradykinin, and leukotrienes. Although this is not ideal, the conclusion that inflammation is neither necessary not sufficient is not particularly weakened. If inflammation were sufficient and the ketoprofen treatment had no effect, then we would expect to see increased *Atf3* expression induced by incisions close-outside of the dermatomal border (i.e., inflammation without axon injury), but this did not occur (Figure 2).

It must be noted that the model used here is one with mixed tissue – the incision injures both the skin and the attached underlying CTM. The exact impact of this is not known, but it is worth mentioning as Brennan and colleagues have reported that incision-related pain is more severe when the damage is to both skin and muscle, as opposed to only skin (28). However, they also note that “…a skin incision without a muscle tissue injury seems to be responsible for inducing mechanical hyperalgesia after incision; muscle injury seems to be not required” (6). One would expect that, if inflammation alone were capable of inducing *Atf3* expression, it would be more likely in the case of incision of both skin and muscle as opposed to skin alone (52, 140), further reinforcing our conclusion that inflammation is not necessary for *Atf3* induction. In terms of clinical relevance, cutaneous muscle coverage is highly variable in humans and is not something that is routinely considered when planning and executing most surgeries, or in follow-up for post-surgical pain.

Although there has been extensive work done to understand the ways that PEG may influence biological systems (21, 22, 69, 70, 72, 156, 159–167), the mechanisms underlying the PEG effects here are unknown. The experimental design was decidedly broad and not refined. Given that a known anti-inflammatory agent did not prevent *Atf3*-induction, but PEG did prevent *Atf3* induction, we presume that PEG is acting through some mechanism other than preventing inflammation (which was shown to be insufficient for inducing *Atf3* expression anyway). The presumption, and the rationale underlying the initial design, is that PEG is acting to enhance membrane integrity and perhaps seal the axolemma injured by the incision. If this were actually occurring it could perhaps prevent or delay some injury signal from causing changes in gene expression at the DRG. In essence, it may be “fooling the neuron into thinking it isn't injured”.

Encouragingly, PEG-treatment also led to a reduction of incision-associated electrophysiological changes, but it is not clear if this is due to prevention of *Atf3*-induction (and thus to the presumed-but-not-proven effects on gene expression), to direct effects on the membrane properties, or both. Importantly, we also examined only a limited time window. PEG effects on *Atf3* expression were examined at 4d (mRNA) and 28d (protein), and electrophysiological properties only at 28d. The reduction of Atf3 at both 4d and 28d is compelling and suggests that the effect of PEG is to prevent *Atf3* induction. It is nonetheless possible that PEG treatment may just delay *Atf3* expression and emergence of electrophysiological sensitization or both, and not fully-prevent it. Fully determining these options will require additional assessments.

We have not yet examined whether autonomic neurons and motor neurons are similarly affected. The focus on sensory neurons is nonetheless highly legitimate given the topic of pain. However, future examination of the other PNS populations is warranted considering the known crossed-influence that can occur, particularly in conditions such as incision and neuroma in which tissue micro-scale compartments can be compromised.

As an aside, for experimental purposes it is possible that PEG-treatment might be a useful way of extending the “acutely-dissociated” status of adult sensory neurons, which start to display injury-associated transcriptional and electrophysiological changes after 8–12 h *in vitro* (varying with species and culture temperature) (32).

Although we did not observe *Atf3* protein expressed in non-neuronal cells, it is certainly possible that this nonetheless occurred either below visual detection threshold or it was expressed at a time we did not examine. Recent work has tied *Atf3* expression by macrophages to persistent neuropathic pain (168). Macrophages infiltrate the DRG and localize near injured sensory neurons after overt nerve injury, but it is not clear if this occurs with incision or other forms of damage to peripheral target tissues. Clarifying this unknown would be very important for determining the relationship of the sensory system response to tissue damage to that of nerve injury.

### Translating animal models to clinical outcomes

“A different paradigm is required for the identification of relevant targets and candidate molecules whereby pain is coupled to the cause of sensorial signal processing dysfunction rather than clinical symptoms” “Given that neuropathic and chronic pain results from a preceding dysfunction in sensory signalling, the identification of effective treatments requires further insight into the reversibility of the underlying dysfunction as well as the timing of intervention relative to the onset of the disease. Novel therapeutic interventions need to be focused at the dysfunction in signalling pathways rather than primarily on pain relief” (169).

Inflammatory and neuropathic pain are rightfully considered different because they result from distinct mechanisms. Inflammatory pain is generally associated with injury to peripheral tissues and neuropathic pain generally associated with injury to the brain, spinal cord, and nerve tissues. Both clinicians and researchers consider the various forms and players of inflammation to be the major cause of persistent pain when there is no obvious damage to a nerve or the CNS [e.g., (1, 2)]. The field also recognizes that there are conditions of tissue pathology with associated pain/itch/dysesthesia of unclear origin, but which are increasingly recognized to have a neuropathic component [e.g., (170)]. The structural and chemical changes in the affected tissues of some of those conditions may induce, with various time-courses, injury to axons resident in that pathologic tissue in addition to any inflammatory processes. We do not debate a role for tissue- or neuro-inflammation in acute or persistent pain. The data presented here, combined with those from others, suggest that damage of peripheral tissues might be inducing additional biological responses and/or conditions that could lead to neuropathic pain, with or without inflammation and inflammatory pain.

**Figure 14:**
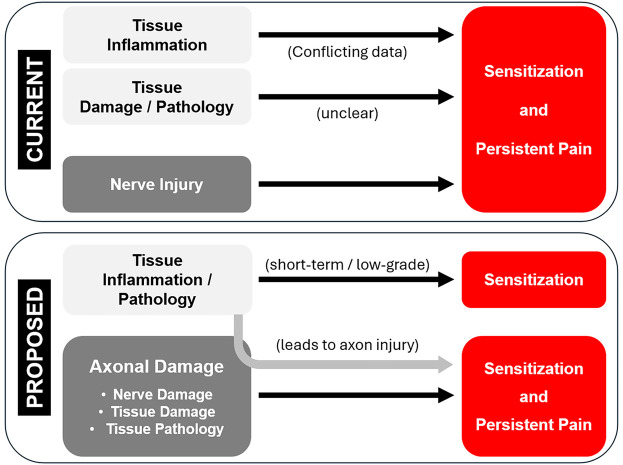
Schematic of the general concept of the relationship of inflammatory and injury conditions to the emergence of sensitization and persistent pain. Current concept is contrasted with the proposed conceptual change. Most notable is inflammation alone would be insufficient to lead to persistent neuropathic pain without concomitant axonal damage, which would unify how nerve injury and tissue damage can lead to persistent neuropathic pain even after wounds are healed and inflammation is resolved or suppressed. This model does not incorporate the many known analgesic/anti-nociceptive mechanisms.

New categories of pain have been introduced, including “nociplastic” (171). This introduction and the pursuant debate [e.g., (172–176)] highlight the need to better understand the mechanisms underlying pain conditions that do not fit into the existing model, and most notably to examine potential new mechanisms. There are features which make it both feasible and attractive to consider these data as a description of a mechanism of “nociplastic pain” [e.g., (177)]. Indeed, the initial proposition for “nociplastic” specifies osteoarthritis as a suitable example condition, and expression of *Atf3* in sensory neurons is induced in animal models of osteoarthritis (12, 14). Nonetheless, we are not advocating for or against the use of any new terms. Instead, we view these and our prior data (9, 10) as supporting a revised consideration of the impact of tissue damage on the nervous system which may simply offer a greater mechanistic understanding of neuropathic pain. This revised consideration could aid in generating a better mechanistic classification of persistent pain after tissue damage. We also lean on many in the field who recognize that there is insufficient mechanistic understanding for how inflammatory processes alone lead to observed phenotypes [e.g., (6, 82)]. In accord with recommendations for using mechanism-based approaches to pain diagnosis and treatment [e.g., (178)], we propose that refining the concept, and recognizing additional biological processes such as we describe here, may better account for the clinical observations and provide a better framework for applying basic research to clinical needs as regards persistent pain after tissue damage and surgery (Figure 14). More work needs to be done to determine if there are meaningful differences in the specific responses to tissue injury-induced axonal damage and nerve injury-induced axonal damage, but the conceptual refinement of considering the long-term consequences of tissue damage as more-similar to nerve injury than to inflammation may provide a significant advance. There are more consequences to tissue damage than inflammation and there can be injury to sensory neurons without damage to discrete nerve tissue. Incorporating these factors into mechanistic and clinical thinking may both add clarity to the classification systems and aid in formulating treatments.

We also used the experimental model and concepts espoused here to provide preliminary indications that simple, feasible, and expandable approaches might be used to prevent some of the biological responses that could be contributing to persistent pain.

### Future directions

The expression of *Atf3* in sensory neurons may serve as a biomarker in experimental settings which can be used to assess a range of chronic pain models for possible contributions due to sensory neuron injury and neuropathic response. Targeting transcription factors as a therapy can be difficult, but this approach for pain control directed to the DRG and spinal dorsal horn already has proof of concept (179). It is possible that directed interference with *Atf3* function in sensory neurons may be an effective approach for preventing and/or treating persistent pain after tissue damage. Assessing the feasibility of this approach will require defining the temporal profile of *Atf3* and other axon injury-associated transcriptional regulators and determine how they interact to control transcription leading to intrinsic sensitization. There is proof of concept for prevention of *Atf3* induction in models of osteoarthritis by pre-treatment with a signaling inhibitor (157) or reduced increase by later administration of an inhibitor of TLR4 (158).

Encouragingly, closing wounds with glue – a common option for many surgeries and injuries – appears to reduce markers of lost-lasting sensory neuron sensitization. This may reduce the risk of developing persistent pain. Further advances in tissue-adhesion/wound-repair may affect the responses reported here. Treating tissue damage with agents like PEG could also become part of the pain-prevention regimen with surgery or trauma (18, 180–186). Although we tested PEG effects with both systemic and topical administration, it is entirely feasible that topical application alone might have similar/suitable effects. Assessing the feasibility of this approach will require directly testing different routes of administration and determining the temporal therapeutic window.

Because there currently is little recognition that “simple” tissue damage, especially when it “heals normally”, might be relevant to a patient's medical history for pain, it will be very difficult to retrospectively construct an accurate estimate of the potential contribution of the response we describe here to persistent pain. Instead, it must be determined prospectively, making it important for the field to consider this biological response alongside other potential contributing factors. We will need research testing to determine if the responses described here exist for humans and animals under our care. If so, we will also need to develop clinical testing procedures and/or biomarkers to determine if these changes are present for those living with pain.

## Data Availability

The original contributions (analyzed data) presented in the study are included in the article/Supplementary Material. Further inquiries can be directed to the corresponding author.
